# 异基因造血干细胞移植治疗BCR::ABL阴性中性粒细胞增殖性肿瘤12例临床研究

**DOI:** 10.3760/cma.j.cn121090-20241211-00560

**Published:** 2025-09

**Authors:** 婷婷 韩, 云 何, 竞 刘, 瑶 陈, 峰蓉 王, 景枝 王, 育红 陈, 海霞 付, 兰平 许, 晓辉 张, 晓军 黄, 昱 王

**Affiliations:** 北京大学人民医院，北京大学血液病研究所，国家血液系统疾病临床医学研究中心，造血干细胞移植治疗血液病北京市重点实验室，北京 100044 Peking University People's Hospital, Peking University Institute of Hematology, National Clinical Research Center for Hematologic Disease, Beijing Key Laboratory of Hematopoietic Stem Cell Transplantation, Beijing 100044, China

**Keywords:** 异基因造血干细胞移植, 不典型慢性粒细胞白血病, MDS/MPN伴中性粒细胞增多, 慢性中性粒细胞白血病, Allogeneic hematopoietic stem cell transplantation, Atypical chronic myeloid leukemia, MDS/MPN with neutrophilia, Chronic neutrophilic leukemia

## Abstract

**目的:**

评价异基因造血干细胞移植（allo-HSCT）治疗BCR::ABL阴性中性粒细胞增殖性肿瘤的疗效及安全性。

**方法:**

纳入2017年3月至2024年6月在北京大学人民医院接受allo‑HSCT治疗的12例中性粒细胞增殖性肿瘤患者，其中慢性中性粒细胞白血病（CNL）8例，骨髓增生异常/骨髓增生性肿瘤伴有中性粒细胞增多（MDS/MPN-N）4例，男7例，女5例，中位年龄48（*IQR*：28，59）岁，对其临床资料进行回顾性分析。

**结果:**

12例患者中6例接受HLA 10/10同胞全相合造血干细胞移植，6例接受单倍体造血干细胞移植。12例患者均获得粒细胞和血小板植入，粒细胞植活中位时间为17（*IQR*：11，24）d，血小板植活中位时间为15（*IQR*：9，28）d。2例患者发生Ⅱ～Ⅳ度急性移植物抗宿主病（GVHD），4例患者发生慢性GVHD。中位随访时间为637（*IQR*：330，943）d，移植后2年总生存率为（65.6±16.4）％，无病生存率为（41.7±16.6）％，累积复发率为（47.2±18.2）％，移植相关死亡率为（11.1±11.4）％。1例患者移植后死于新型冠状病毒肺炎，2例死于复发。

**结论:**

allo-HSCT是BCR::ABL阴性中性粒细胞增殖性肿瘤安全、有效的治疗方法。

## Abstract (en)

**Objective:**

To investigate the efficacy and safety of allogeneic hematopoietic stem cell transplantation（allo-HSCT）for the treatment of BCR::ABL-negative chronic neutrophilic leukemia（CNL）and MDS/MPN with neutrophilia.

**Methods:**

This study retrospectively analyzed 12 cases of CNL and MDS/MPN with neutrophilia that underwent allo-HSCT from March 2017 to June 2024, comprising 7 males and 5 females with a median age of 48（*IQR*:28, 59）years. The 2-year overall survival（OS）, disease-free survival（DFS）, cumulative incidence of relapse（CIR）, and transplantation-related mortality（TRM）rates were analyzed. Complications were also assessed.

**Results:**

Of the 12 patients, 6 received matched sibling HSCT and 6 received haploidentical HSCT. All patients had successful engraftment, and the median times of neutrophil and platelet engraftment were 17（*IQR*:11, 24）days and 15（*IQR*:9, 28）days, respectively. Grade Ⅱ–Ⅳ acute graft versus host disease（GVHD）and chronic GVHD occurred in 2 and 4 cases, respectively. The 2-year OS, DFS, CIR, and TRM rates were（65.6±16.4）％,（41.7±16.6）％,（47.2±18.2）％, and（11.1±11.4）％, respectively, after a median follow-up time of 637（*IQR*:330, 943）days. One patient died from treatment-related complications due to respiratory failure caused by coronavirus disease 2019. Two patients died due to relapse.

**Conclusion:**

Allo-HSCT can be applied as a safe and effective approach to treat CNL and MDS/MPN with neutrophilia.

慢性中性粒细胞白血病（Chronic neutrophilic leukemia, CNL）和不典型慢性髓性白血病（aCML）［2022年WHO诊断标准命名为骨髓增生异常/骨髓增生性肿瘤伴有中性粒细胞增多（MDS/MPN-N）］都是罕见的BCR::ABL阴性骨髓增殖性肿瘤（Myeloproliferative neoplasm, MPN）[1]–[2]，均以外周血中性粒细胞持续升高为主要表现，常伴有脾大，并且具有与其他骨髓肿瘤相同的某些基因突变（SETBP1、ASXL1、U2AF1、SRSF2和TET2等）[3]–[4]。CNL转化为白血病的概率为10％～25％，中位生存期15～31个月[4]–[5]；MDS/MPN-N转化为白血病的概率为30％～40％，中位生存期12～20个月[6]–[10]。现有的治疗方法（羟基脲、干扰素、JAK抑制剂、去甲基化药物及化疗）仅能通过控制细胞改善临床症状，并不能改变疾病进程[2]。由于这两种疾病的发病率较低，异基因造血干细胞移植（allo-HSCT）治疗该疾病的相关报道也极少。本研究对在本中心接受allo-HSCT的CNL、MDS/MPN-N患者进行回顾性分析，旨在评估allo-HSCT治疗该类疾病的安全性及有效性。

## 病例与方法

1. 病例：本研究为回顾性队列研究，纳入2017年3月1日至2024年6月30日期间在北京大学人民医院血液科接受allo-HSCT的CNL和MDS/MPN-N患者。

2. 诊断标准：采用2022年WHO诊断标准[11]。

3. allo-HSCT方案：同胞全相合移植采用改良白消安（Bu）/环磷酰胺（Cy）预处理方案；单倍体移植及无关供者移植均采用改良Bu/Cy+抗胸腺细胞球蛋白（ATG）方案。移植物为rhG-CSF动员后的骨髓联合外周血干细胞。采用环孢素A（CsA）、霉酚酸酯（MMF）及短程甲氨蝶呤（MTX）方案预防急性移植物抗宿主病（GVHD），同胞全相合移植且年龄>40岁的患者加用小剂量ATG预防GVHD。常规予阿昔洛韦、复方磺胺甲噁唑及抗真菌药物预防病毒、卡氏肺孢菌及真菌感染[12]。

4. 定义：中性粒细胞植活：连续3 d中性粒细胞绝对计数（ANC）>0.5×10^9^/L；血小板植活：连续7 d血小板计数>20×10^9^/L且脱离血小板输注。急性GVHD的诊断与预防方案及分度参照《中国异基因造血干细胞移植治疗血液系统疾病专家共识（Ⅲ）急性移植物抗宿主病（2024年版）》[13]。慢性GVHD的诊断与预防方案诊断及分度参照《慢性移植物抗宿主病诊断与治疗中国专家共识（2024年版）》[14]。CNL和MDS/MPN-N的疗效标准参照2015年成人国际MND/MPN治疗疗效评价标准[15]。

5. 随访：随访数据来自住院/门诊病历和电话随访记录。随访截止日期为2024年10月20日。

6. 统计学处理：采用SPSS 26.0软件对数据进行统计分析。计量资料采用ShaPiro-Wilk进行正态性检验，正态分布计量资料以*x*±*s*表示，偏态分布计量资料采用*M*（*Q*1，*Q*3）表示，计数资料以频数及百分率表示。使用Kaplan‑Meier方法绘制生存曲线，移植相关死亡率（TRM）或复发采用竞争风险模型进行评估。以*P*<0.05为差异有统计学意义。

## 结果

1. 病例特点：本项回顾性研究纳入2017年3月至2024年6月间在北京大学人民医院血液病研究所诊断为CNL或MDS/MPN-N并接受allo-HSCT的12例患者，其中CNL 8例，MDS/MPN-N 4例，男7例，女5例，中位年龄48（28，59）岁。12例患者移植前血常规（中位数）：WBC 48.5（23，120）×10^9^/L，HGB 104（40，130）g/L, PLT 115（40，349）×10^9^/L，从诊断到移植的中位时间为8.1（2.9，25.9）个月。8例CNL患者中，5例移植前处于慢性期，3例处于加速期。4例MDS/MPN-N患者中1例移植前处于加速期，其余3例处于慢性期；移植前治疗情况详见表1。

**表1:** 12例BCR::ABL阴性中性粒细胞增殖性肿瘤患者一般资料

例号	诊断	分期	基因突变	脾脏	治疗	移植前疾病状态
1	CNL	慢性期	CSF3R、U2AF1、SETBP1	正常	羟基脲	慢性期
2	CNL	慢性期	CSF3R、SETBP1、DNMT3A、ASXL1	正常	DA方案	加速期
3	CNL	慢性期	CSF3R	正常	羟基脲	慢性期
4	CNL	慢性期	CSF3R、ASXL1、SETBP1、ZRSR2、KMT2B	肋缘下3.8 cm	干扰素	慢性期
5	CNL	慢性期	CSF3R	正常	羟基脲	加速期
6	CNL	加速期	CSF3R、DNMT3A、SETBP1、U2AF1	正常	羟基脲	慢性期
7	CNL	慢性期	CSF3R、ASXL1、NRAS、SETBP1	肋缘下平脐	芦可替尼	慢性期
8	CNL	急变期	CSF3R、ASXL1	正常	羟基脲、DA方案	加速期
9	MDS/MPN-N	慢性期	ASXL1、SETBP1	正常	羟基脲、干扰素	慢性期
10	MDS/MPN-N	慢性期	ASXL1、SETBP1、SRSF2、CBL	正常	羟基脲	慢性期
11	MDS/MPN-N	慢性期	ASXL1、SETBP1、NRAS、PTPN11、SF3B1	肋缘下3.9 cm	羟基脲	慢性期
12	MDS/MPN-N	慢性期	ETV6、SETBP1、U2AF1	正常	地西他滨	加速期

**注** CNL：慢性中性粒细胞白血病；MDS/MPN-N：骨髓增生异常/骨髓增生性肿瘤伴有中性粒细胞增多；DA方案：柔红霉素+阿糖胞苷

12例患者中6例接受同胞全相合移植，6例接受单倍体移植，中位单个核细胞输注量为9.19（6.56，13.17）×10^8^/kg，中位CD34^+^细胞输注量为2.90（1.72，21.18）×10^9^/kg。

2. 造血重建和嵌合状态：12例患者均获得粒细胞和血小板植入，粒细胞植活中位时间17（11，24）d，血小板植活中位时间15（9，28）d。例7移植后78 d感染新型冠状病毒（新冠）肺炎后出现继发性植入功能不良，经过促造血治疗后血象逐渐恢复，DNA指纹图为完全供者型，原发病评估为缓解状态。所有患者移植后通过骨髓性染色体荧光原位杂交检测或聚合酶链反应扩增短串联重复序列方法监测供受者嵌合状态，例2和例11移植后1个月DNA指纹图显示为混合嵌合，其余患者移植后评估均为完全供者型。除例2和例11外，3例复发的患者在复发当时检测DNA指纹图为混合嵌合状态。

3. 复发及生存情况：12例患者移植后均获得CR。其中5例发生复发。例2于移植后1个月流式细胞术微小残留病检测（FCM-MRD）0.08％，DNA指纹图为混合嵌合状态；1.5个月出现全血细胞减少，骨髓原始细胞4％，FCM-MRD 0.44％，考虑复发，给予AA方案（阿柔比星、阿糖胞苷）化疗和供者淋巴细胞输注（DLI）；移植后3个月复查骨穿FCM-MRD 0.13％，STR仍显示混合嵌合，给予IFN-α治疗后出现慢性GVHD，之后监测患者MRD持续阴性，STR为完全供者型，血象恢复。例3于移植后2个月骨髓原始细胞5％，FCM-MRD 1.18％，WT1和PRAME升高，给予VA方案［维奈克拉（Ven）+阿扎胞苷］化疗及DLI治疗无效，行二次单倍体移植（父供子）后MRD持续阴性，患者合并重度cGVHD。例5于移植后3个月骨髓原始细胞12％，提示复发，予以AA方案化疗及DLI，地西他滨+Ven及索拉菲尼+Ven治疗均无效，患者死于复发。例11移植后1个月混合嵌合，移植后60 d出现全血细胞减少，外周血常规示原始粒细胞1％、中晚幼粒细胞19％，DNA指纹图为混合嵌合，提示复发，阿扎胞苷+Ven方案化疗及DLI治疗无效，然后采用二次单倍体移植（父供子），目前移植后尚未评估；例12移植后13个月FCM-MRD 1.44％，WT1及PRAME升高，HAA（高三尖杉酯碱+阿糖胞苷+阿柔比星）方案化疗及DLI治疗无效，再次给予阿扎胞苷+Ven方案化疗仍无效，死于复发（表2）。

**表2:** 12例BCR::ABL阴性中性粒细胞增殖性肿瘤患者allo-HSCT相关资料及疗效

例号	预处理方案	供者	移植模式	干细胞来源	急性GVHD	慢性GVHD	移植后疗效	复发	随访结局	OS（d）
1	BU/CY/FLU+ATG	子	单倍体	外周血	Ⅱ度	轻度（皮肤）	CR	否	存活	555
2	BU/CY+ATG	兄	同胞全相合	外周血	/	轻度（皮肤）	CR	是	存活	637
3	BU/CY	兄	同胞全相合	外周血	/	重度（肺）	CR	是	存活	894
4	BU/CY+ATG	女	单倍体	外周血	/	重度（肝+肺）	CR	否	存活	396
5	BU/CY	妹	同胞全相合	外周血	/	/	CR	是	死亡	259
6	BU/CY+ATG	姐	同胞全相合	外周血	/	/	CR	否	存活	152
7	BU/CY/FLU+ATG	子	单倍体	骨髓+外周血	/	/	CR	否	存活	135
8	BU/CY+ATG	女	单倍体	骨髓+外周血	/	中度（皮肤+口腔）	CR	否	存活	2 783
9	BU/CY+ATG	妹	同胞全相合	外周血	/	/	CR	否	存活	865
10	BU/CY/FLU+ATG	女	单倍体	外周血	/	/	CR	否	死亡	156
11	BU/CY	姐	同胞全相合	外周血	/	/	CR	是	存活	211
12	BU/CY+ATG	子	单倍体	骨髓+外周血	Ⅲ度	/	CR	是	死亡	506

**注** allo-HSCT：异基因造血干细胞移植；GVHD：移植物抗宿主病；BU：白消安；CY：环磷酰胺；FLU：氟达拉滨；ATG：抗胸腺细胞球蛋白；CR：完全缓解；OS：总生存；/：无

中位随访时间为637（330，943）d。至随访截止，9例患者处于无病生存状态，2例复发死亡（图1），移植后2年总生存（OS）率为（65.6±16.4）％，无病生存（DFS）率为（41.7±16.6）％，累计复发率（CIR）为（47.2±18.2）％。

**图1:**
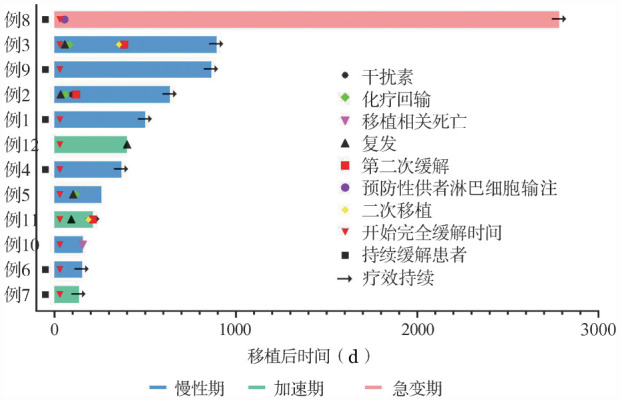
12例BCR：：ABL阴性中性粒细胞增值性肿瘤患者治疗及生存情况

4. GVHD和移植相关并发症：2例患者发生Ⅱ～Ⅳ度急性GVHD，1例患者经糖皮质激素治疗后好转，另1例患者发生难治性急性GVHD，经CD25单抗等联合治疗后好转。5例患者发生慢性GVHD，其中2例为重度，1例为中度，另外2例为轻度。3例患者分别于移植后33、31和41 d发生巨细胞病毒（CMV）血症，均经过更昔洛韦抗病毒治疗后好转；2例患者分别于移植后28 d和62 d发生EB病毒相关淋巴细胞增殖性疾病（PTLD），经过CD20单抗治疗后好转。本组患者中仅1例患者移植后合并新冠肺炎死亡，2年TRM为（11.1±11.4）％。

## 讨论

CNL和MDS/MPN-N由于发病率低、缺乏特异性诊断标志，具有较高的转化为急性白血病的风险，OS期短，预后差。现有的治疗方式包括非移植治疗和allo-HSCT。非移植治疗的目的主要是缓解临床症状，提高生活质量。allo-HSCT是唯一能治愈该疾病的治疗手段，但是现有数据有限，大宗病例报告极少。

自1996年Hasle等[16]报道2例CNL患者经过移植获得长期缓解后，陆续有小宗病例报告[17]–[19]。关于移植，首先需要评估的是哪些患者需要进行？国内肖志坚教授团队回顾性分析16例CNL患者的临床和实验室特征及预后因素，初诊时WBC>50×10^9^/L的患者中位OS期仅为11个月，是预后不良的指标[5]。有研究认为合并ASXL1突变及血小板减少的患者预后差[20]。来自美国梅奥中心19例CNL患者的数据显示：中性粒细胞计数>60×10^9^/L、PLT<160×10^9^/L、ASXL1突变是预后不良的因素，基于这些指标，将患者分为高危和低危，建议高危患者进行更严密的监测和尽早行allo-HSCT[21]。近期日本一项多中心研究显示，移植前处于加速期或急变期的患者移植预后更差，而处于慢性期的患者可获得更好的生存[22]。本研究中8例CNL患者中3例移植前处于加速期，其中2例行同胞全相合移植的患者均发生移植后早期复发，1例通过化疗、DLI及干扰素治疗获得无病生存，另1例化疗、DLI无效并死亡；另外1例行单倍体移植的患者，移植后给予预防性DLI治疗后获得无病生存。5例患者移植前处于慢性期，其中1例接受同胞全相合移植的患者发生移植后早期复发，进行二次单倍体移植后获得无病生存。从我们的数据来看似乎在慢性期接受移植的患者移植预后较好，与文献报道一致。本研究中，4例接受单倍体移植的患者均获得无病生存，而4例接受同胞全相合移植的患者中有3例复发，似乎单倍体移植有更好的治疗效果。

Koldehoff等[23]报道9例aCML患者接受allo-HSCT，中位随访55个月后有8例患者存活。之后又报道21例aCML患者移植后5年OS率为80％[24]。一项来自EBMT的研究结果显示，42例患者移植后5年无复发生存（RFS）率为36％、CIR为40％，TRM为24％[25]。可见allo-HSCT可明显改善aCML患者的预后[26]，越来越多的患者选择该治疗[8]。

目前关于MDS/MPN-N患者的移植时机尚无共识，有学者认为诊断后符合条件的患者应该尽早接受移植，也有学者认为应该根据诊断后的危险分层建议中高危的患者接受移植。梅奥中心的25例aCML患者的数据显示年龄>67岁、HGB<10 g/L和合并TET2突变是预后不良因素，根据这三项指标将患者区分为低危（中位OS期18个月）和高危（中位OS期7个月）[7]。一项纳入65例MDS/MPN-N患者的研究中显示：年龄、骨髓原始细胞比例、血小板计数和LDH水平是影响生存的危险因素，把这几项因素纳入后形成一个预测生存的模型来估算其生存，分数越高生存越差[26]。日本学者Itonaga等[22]报道的19例患者（aCML 15例，CNL 4例）的分析显示外周血原始细胞≥5％是预后不良的因素。因此，这些研究提示我们应该对具有高危因素的患者进行allo-HSCT。我们中心这4例接受allo-HSCT的患者3例处于慢性期进行allo-HSCT，其中1例获得无病生存，1例死于新冠肺炎，另外1例移植后早期复发，化疗及DLI无效，接受二次移植治疗。另外1例加速期的患者移植后死于复发。近期苏州大学附属第一医院报告31例aCML患者，采用去甲基化为基础方案治疗组的OS率33％，而allo-HSCT组中位OS期仅为20个月，因此提出allo-HSCT是否能改善预后需要重新评估[27]。

本研究12例患者均获得粒细胞植入，中位植入时间17（11，24）d，与文献[22]报道的20（15～29）d和文献[23]报道的16（8～21）d基本一致。本中心既往报告伴脾肿大慢性粒-单核细胞白血病患者中位粒细胞植入时间为17（11～20）d[28]，推测可能与部分MPN患者伴有脾脏增大导致干细胞归巢延迟或者干细胞被破坏导致植入延迟有关。既往有研究发现由于GVHD和感染导致较高的TRM，而本研究中患者移植TRM与我们既往数据一致，提示allo-HSCT对这部分病例是安全可行的。

综上，allo‑HSCT是目前可能治愈CNL和MDS/MPN-N的方法，复发仍然是该类疾病移植后死亡的主要原因，对这些患者进行全面、系统的预后评估并制定个体化诊疗方案可能使更多患者获益，最佳移植时机、预处理方案、供者选择及移植后预防复发都是尚待解决的问题。
